# Review on Hydrogel-Based Flexible Supercapacitors for Wearable Applications

**DOI:** 10.3390/gels9020106

**Published:** 2023-01-26

**Authors:** Melkie Getnet Tadesse, Jörn Felix Lübben

**Affiliations:** 1 Sustainable Engineering (STE), Albstadt-Sigmaringen University, 72458 Albstadt, Germany; 2 Ethiopian Institute of Textile and Fashion Technology, Bahir Dar University, Bahir Dar 1037, Ethiopia

**Keywords:** flexible supercapacitors, wearable electronics, hydrogels, conductive polymers

## Abstract

Smart hydrogels with high electrical conductivity, which can be a real source of power while also collecting and storing the diverse sources of energy with ultrahigh stretchability, strong self-healability, low-temperature tolerance, and excellent mechanical properties, are great value for tailored wearable cloths. Considerable effort has been dedicated in both scientific and technological developments of electroconductive hydrogels for supercapacitor applications in the past few decades. The key to realize those functionalities depends on the processing of hydrogels with desirable electrochemical properties. The various hydrogel materials with such properties are now emerging and investigated by various scholars. The last decade has witnessed the development of high-performance supercapacitors using hydrogels. Here, in this review, the current status of different hydrogels for the production of flexible supercapacitors has been discussed. The electrochemical properties such as capacitance, energy density and cycling ability has been given attention. Diverse hydrogels, with their composites such as carbon-based hydrogels, cellulose-based hydrogels, conductive-polymer-based hydrogels and other hydrogels with excellent electromechanical properties are summarized. One could argue that hydrogels have played a central, starring role for the assembly of flexible supercapacitors for energy storage applications. This work stresses the importance of producing flexible supercapacitors for wearable clothing applications and the current challenges of hydrogel-based supercapacitors. The results of the review depicted that hydrogels are the next materials for the production of the flexible supercapacitor in a more sustainable way.

## 1. Introduction

Hydrogels are often known as three-dimensional networks of polymer chains capable of holding a large quantity of water while also being water insoluble. Research into “hydrogels” is currently a growing topic of scientific interest due to their diverse applications and eco-friendly properties. Hydrogels have broad applications such as drug delivery [1], tissue engineering [2], wound healing [3], hygiene product development [4] and supercapacitors’ development [5]. During the last few decades, the research and development in the area of wearable clothing has been dynamically increasing. However, the challenge in the power supply is still remains a challenge. Due to this fact, a lightweight, flexible, durable, with excellent electro-mechanical properties battery source is avoidably required [6]. All the components in wearable clothing are required to provide functionality and comfortability (weight, tactile sensation, thermal comfort, flexibility and stretchability) to the wearer during service times [7]. Hydrogels are unique due to its bio-compatibility, higher content and are often used as electrolyte materials [8]. Therefore, we require hydrogels in greater demand.

Smart textiles have diverse applications such as heat storage [9], communications devices [10], piezoelectric energy harvesting [11], thermoelectric energy harvesting [12], electroluminescence applications [13], energy storage [14] and thermoregulated clothing [15]. For the aforementioned applications, sustainable production of the power source is strongly fundamental. There are many ways that energy storage can be achieved. These energy storages can be achieved by various mechanisms such as thermal [16], battery-based [17], an electrochemical energy storage device [18], and supercapacitor energy storage devices [19]. Among the list given above, supercapacitor-based energy storage devices have been given attention in recent times [18]. This is because not only supercapacitors provide broad applications such as in hybrid vehicles, smartphone components and energy harvesting devices but also deliver superior performance [20]. Supercapacitors can be made real by using different approaches using self-charging power textiles [21], or incorporating conducting materials into substrates such using graphene (GO) [22], poly propylene (PPy) [23], poly(3,4-ethylenedioxythiophene):poly(styrenesulfonate) (PEDOT:PSS) [24] and functional hydrogels [25].

Functional hydrogels are called next generation materials for the production of supercapacitors [26]. This is because hydrogels are flexible and have unique features to be used in wearable applications [27]. In this regard, ionic-based hydrogels have been implemented for high energy storage applications [28].The hydrogels are sustainable and environmentally friendly; easy processability made them to be assumed the next generation of materials used in several application fields. This review work addressed and critically revised hydrogel-based supercapacitors for energy storage applications.

## 2. Hydrogels

**Figure 1:**
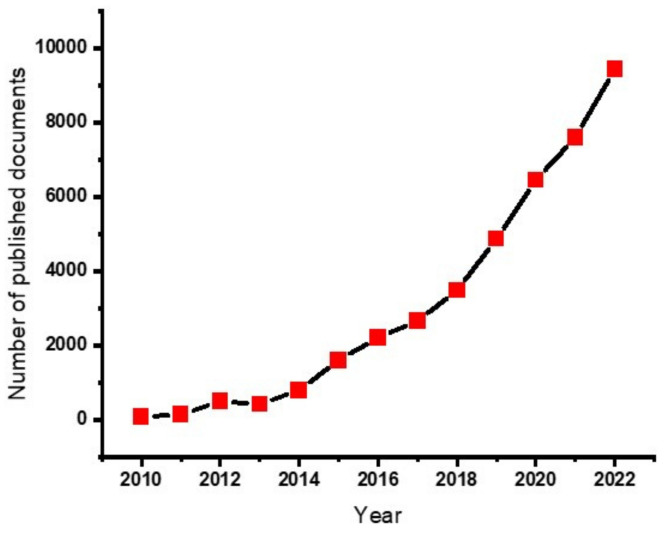
Number of published papers on the https://app.dimensions.ai/ data base with the search of “hydrogels for supercapacitor” (the data is accessed on 20 January 2023).

Non-renewable fossil fuel-based energy sources are still commonly encountered and remain a challenge in sustainable environment. Scholars all over the world are struggling to replace them, using sustainable means to protect the environment from the pollution caused by emissions from the fossil fuels. One of the solutions could be using supercapacitors for energy storage applications from bio-based products. Supercapacitors are light in weight and flexible, and can charge faster, are more durable, and can be used in wearable applications [29]. The development of supercapacitors based on hydrogel materials demonstrated a dynamic growth in the research arena. Figure 1 illustrates how the development grows rapidly each year.

The publication record gives an indication that the use of hydrogels for the production of hydrogels is increasing and can be a future of energy storage applications.

**Figure 2:**
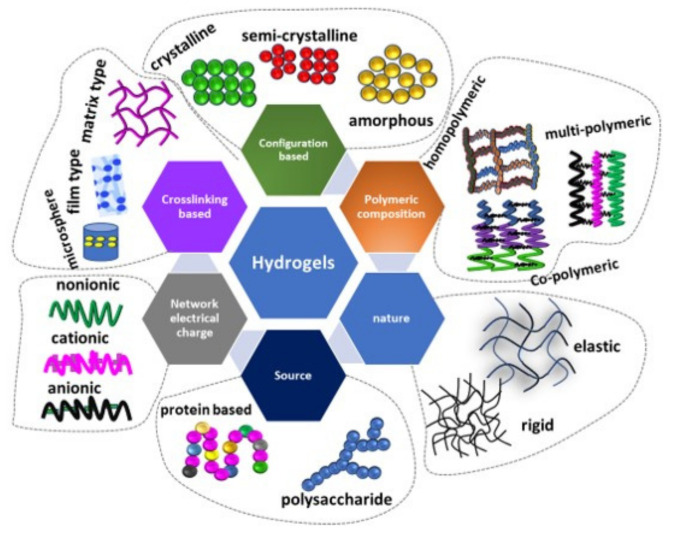
Review on the different classification of hydrogels.

Hydrogel-based electrolytes demonstrated a promising result in supercapacitors’ applications. The hydrogels are highly hydrophilic substances with well-defined structures and do not dissolve in water. Hydrogels can be obtained from various sources, such as macromolecules extracted from animal collagen [30], plants [31] and seaweed [32]. The basic foundation for the hydrogel is polysaccharides and proteins with fundamental constitutes of glycosidic and amino acid repeating units, respectively. The basic properties of the hydrogel is capturing very huge amounts of water molecules in its 3D networks [33]. Hydrogels can be classified based on polymeric composition, configuration, types of crosslinking, physical appearance and electrical charge [34], as summarized in Figure 2.

**Figure 3:**
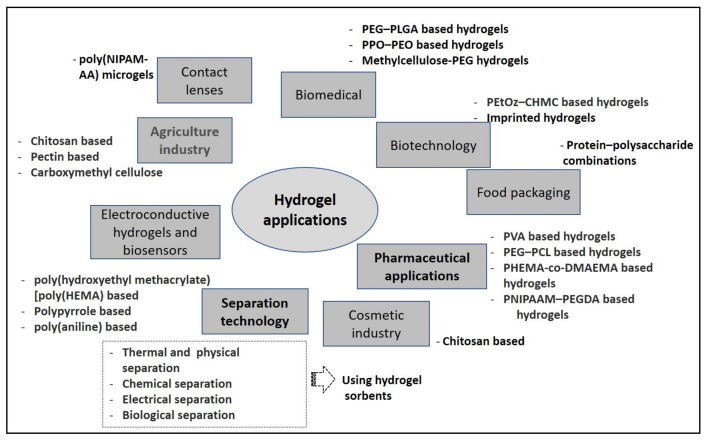
Various applications of hydrogels. The texts were extracted from Ref. [35].

The critical properties of hydrogel material are that it is highly sensitive to the environment, as it is hydrophilic polymers. It can swell and de-swell in water and can keep a large volume of water in swollen state [34]. This makes hydrogel materials sensitive to environmental stimuli such as electrical, temperature, sound and ionic substances. Due to this fact, hydrogel materials are perfect candidates for several applications. Figure 3 shows various application areas for hydrogel materials.

**Figure 4:**
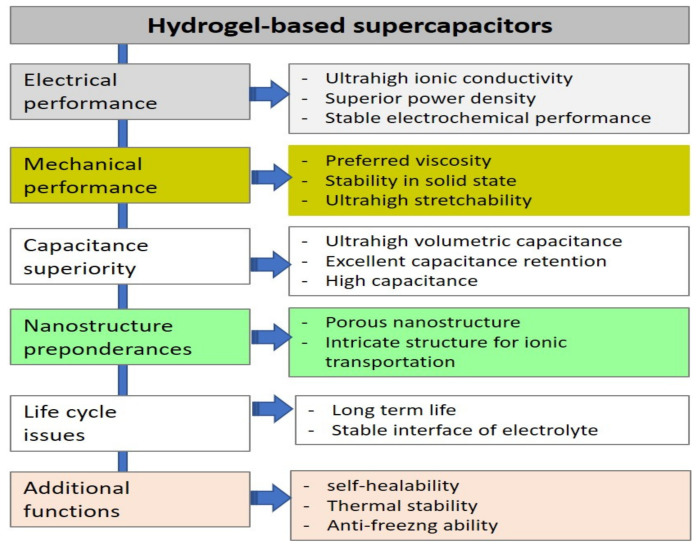
Reasons why hydrogel-based supercapacitors are preferred. Re-produced with permission with Under a Creative Commons (CC-BY) license 4.0 with Ref. [37].

This review focuses on one of the applications of hydrogels, which is the electroconductive hydrogel. Electroconductive hydrogels are among the conductive materials that can be used for smart applications. One of the functional applications of electroconductive hydrogels is for supercapacitor applications [36]. Hydrogel-based supercapacitors are self-healable, stretchable, have excellent mechanical performance and other several performances to be selected [37]. Figure 4 shows the various perspectives of supercapacitors from hydrogel-based materials.

All the above-mentioned properties make hydrogel a perfect candidate to produce high performance supercapacitors with superior flexibility, durability, high energy and power density, and high charge-discharge capacity, which is further used for the production of smart clothing applications. In the following section, various hydrogels that can be used for the production of supercapacitors, which will be used to manufacture/assemble smart clothing, are discussed.

## 3. Hydrogels for Supercapacitor Production

The increase in the demand of energy storage materials provide an opportunity of using supercapacitors to be used for wearable clothing applications. In this regard, hydrogel-based supercapacitors are new emerging technologies for wearable electronics due to its flexibility, stretchability and lightweightdness properties [25]. Various conducting polymer-based hydrogels demonstrate great potential for supercapacitor applications due to excellent electrical conductivity, flexibility, electrolytic properties, excellent solid–liquid interface and self-healing ability [38]. This section provides various insights on the various hydrogel electrolytes for supercapacitor application where further used for the production of wearable electronics. High efficiency, with high energy density energy storage materials for wearable electronics, is seldom required. The applications of hydrogels in the supercapacitor can be either as an electrode or electrolyze or can be both at the same time. The general applications of hydrogels in supercapacitor production are detailed in this review. Hydrogel-based materials will solve the problems created by other types of supercapacitors’ materials. Hydrogels are highly resistance to external environmental conditions and have good mechanical properties.

### 3.1. Electrochemical Properties of Hydrogels

Hydrogels continue to be used in potential applications for supercapacitor production due to their bio-compatibility [39]. In addition to their bio-compatibility, their electrochemical performance plays an important role in producing energy storage materials [40]. In order to measure the electrochemical properties of hydrogels, first the hydrogel shall be converted into electrodes. The electrodes can then be characterized by measuring the electrical conductivity of the electrode material first hand to observe whether the material possesses electrical conductivity. The electrical conductivity can be measured using four probe principles [41]. Then, the most applicable way of measuring the electrochemical performance of the hydrogels is measuring the specific capacitance, and the charge-discharge capability at various current densities [39]. The most important equipment to measure the electrochemical characteristics of the hydrogel is the electro impedance spectroscopy (EIS). It is familiar that EIS can help to demonstrate potentiostats and galvanostats, which measure the cyclic voltammetry (CV) characteristics and charge-discharge capability of the hydrogel materials. Meng, X., et al. stated that the galvanostatic charge–discharge testing results in various current densities and specific capacitance using potentiostats’ principles. Furthermore, the authors have used Nyquist plots to explore the electrochemical kinetics of the electrodes. In general, the electrochemical measurements have been measured almost in similar manner, which is using EIS instruments.

### 3.2. Carbon-Based Hydrogels

The incorporation of carbon-based nanomaterials in the production of hydrogels has been given recent attention for various applications. Carbon-based hydrogels can be further classified into graphene-based hydrogels, polymer-based hydrogels and bio-mass-derived hydrogels [42]. These hydrogels are carbon-based [43] because they comprise carbon in their constituent compound, and they can be used for the production of supercapacitors, as they are conductive in nature. Carbon-based hydrogels from natural resources provide an option to obtain the sustainable manufacturing of supercapacitors [44].

#### 3.2.1. Graphene-Based Hydrogels

**Figure 5:**
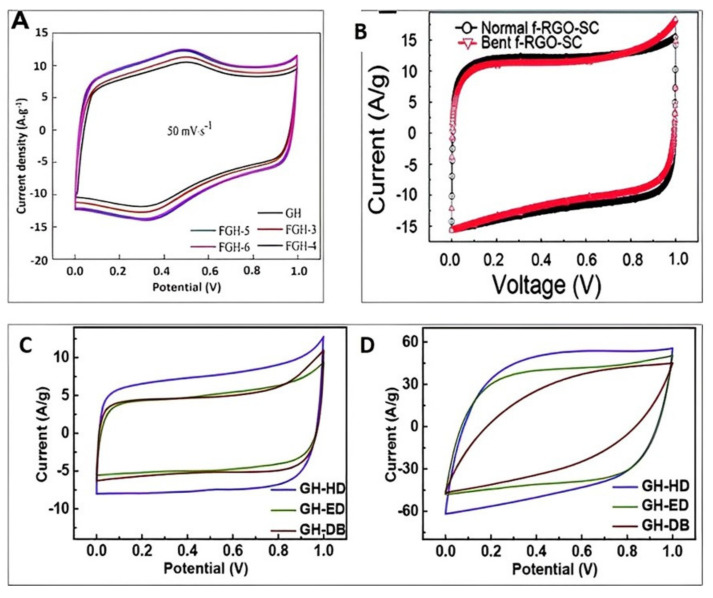
Electrochemical performance of supercapacitors based on graphene hydrogels. (**A**) charge-discharge characteristics of graphene-based electrode at a current density of 1 Ag^−1^ and scan rate of 50 mV/s; re-printed with an open access article under the terms of CC BY with Ref. [45]. (**B**) Cyclic voltammograms at a 100 mV/s scan rate of a flexible-RGO-supercapacitor before and after bending; reproduced with permission [47]. Copyright © 2011, American Chemical Society. (**C,D**) Cyclic voltammograms of gels obtained at scan rates of (**C**) 100 mV/s and (**D**) 1000 mV/s in the voltage range of 0–1 V; reproduced with permission with License Number: 5447060051277 [48]. Copyright © 2017, Journal of Power Sources, Elsevier.

Graphene-based hydrogels are perfect candidates for the synthesis of supercapacitors due to their excellent rate capabilities, ultrahigh energy density and durability. Ju et al. [45] have reported the preparation of very high performance supercapacitors from ethylenediamine (EDA) functionalized graphene hydrogels (FGHs) as electrode materials. The specific energy, cycle life and capacity are very important parameters required for the production of high-performance supercapacitors. The fundamental properties of materials to be used in supercapacitor applications is its electrochemical performance. Figure 5 demonstrates the various studies on the electrochemical performance of graphene-based supercapacitors. Graphene-based hydrogels, with PVDF as an electrolyte, demonstrated excellent supercapacitor performance [46] on paper substrates.

For the ultrahigh efficiency of supercapacitor applications, the base materials have to possess high porosity, very high surface area, excellent mechanical properties and fast electron transfer. Due its three-dimensional architectures, graphene-based hydrogels fulfil these fundamental properties [49]. The preparation method has a great influence on its final output. Chen et al. [50] reported the preparation of graphene-based hydrogels with nickel hydroxide and obtained a capacitance as high as 3138.5 F. This value is very sufficient to store energy and is equivalent to the theoretical capacitance. This implies that hybrid hydrogels using graphene are excellent materials for supercapacitor applications. Furthermore, Table 1 depicts the various capacitance values obtained using graphene-based hydrogels. Current density values are incorporated for comparison purposes.

Utilizing 3D architectural graphene structures for the production of supercapacitors using various mechanisms with different composite materials provides excellent performance in terms of specific capacitance value. Graphene-based hydrogels received tremendous attention in recent times for such applications. Flexibility, high energy density, and superior capacitance with acceptable charge-discharge capability demonstrate a promising performance. Based on the review, graphene-based hydrogels can be used as electrode and electrolyte materials for supercapacitor applications with excellent electrochemical performance.

#### 3.2.2. Carbon Nanotubes

**Figure 6:**
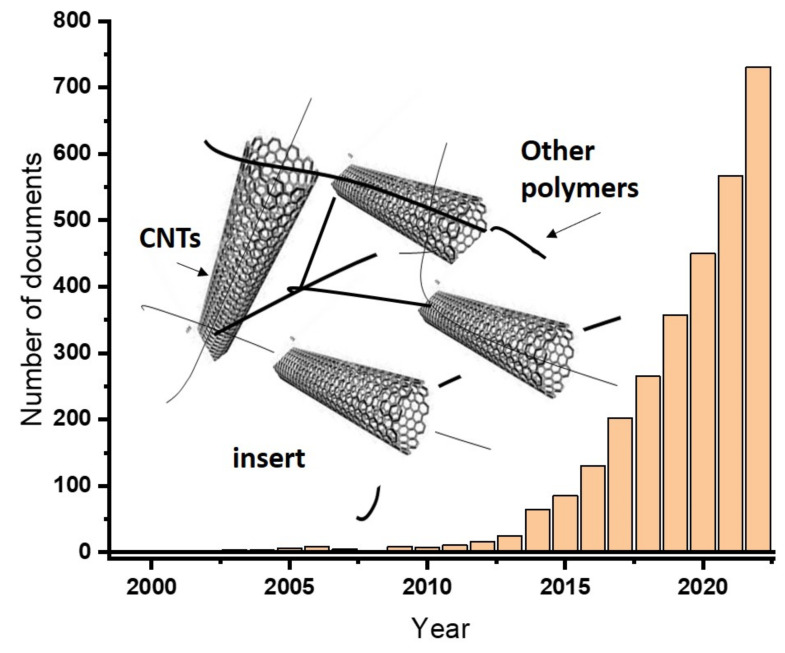
The increasing trends on the use of carbon nanotubes in the production of supercapacitors searched on the topic of “carbon nanotube-based hydrogels for supercapacitor applications” (as obtained from ScienceDirect databases (accessed on 12 December 2022). The insert representing the hydrogels is formed by CNTs.

Carbon-based nanomaterials possess excellent and exceptional mechanical strength, ultrahigh electrical conductivity, high specific surface area and sole hierarchical structures [65]. Due to the above-mentioned facts, carbon-based nanomaterials are excellent candidates for supercapacitor applications. Carbon-nanotubes (CNTs) are carbon nanomaterials with no exception for such applications. Recent publications indicated that supercapacitors based on CNT is growing. Figure 6 shows the growing trends of the use of CNTs hydrogels for the production of supercapacitors. Carbon nanotubes’ (CNTs) electrodes have been fabricated using polyvinylidene fluoride (PVDF) and polyvinyl alcohol (PVA) as a gel electrolyte, which help to obtain a capacitance value of 173 F/g [66].

The preparation of supercapacitors using CNTs also includes the formation of different composites using different polymers. Table 2 shows the performances of supercapacitors produced from CNT-based hydrogels incorporating various polymers as a composite element. The result depicts exceptional performances. Current density (A/g; mA/cm^2^) and power density (mW cm^−3^) values are incorporated for comparison purposes.

### 3.3. Conductive Polymer-Based Hydrogels

Hydrogels based on conductive polymers are materials that contain conductive polymers within a cross-linked network structure of the electrically insulative polymer gel [75] and conductive network hydrogels [76]. The electrically conducting polymer hydrogels can be produced using various polymerization techniques depending on the types of monomers, and the conducting polymer hydrogels are further classified depending on the polymer type. This section discusses the use of different conducting polymers for the preparation of supercapacitors.

#### 3.3.1. PAMPS-Based Hydrogels

**Figure 7:**
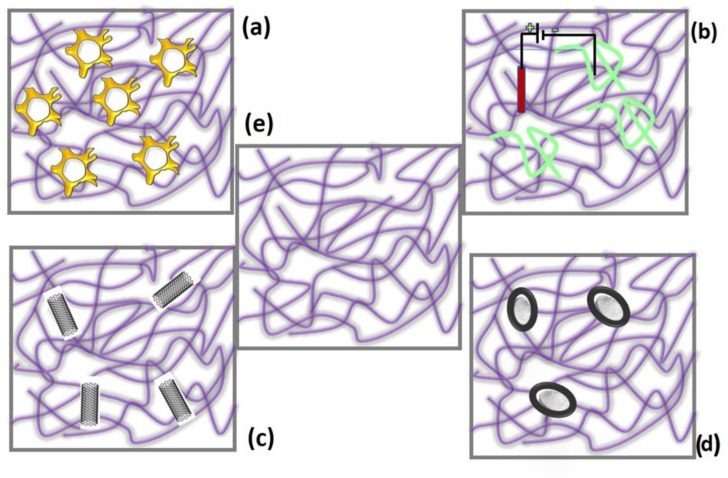
Various composite polymers that can form excellent compatibility with PAMPS for supercapacitor applications. (**a**) Dopant in 3D polypyrrole network formed by synergetic coupling with graphitic oxide and obtaining highest capacitance of 698.8 F g^−1^ at 5 mV s^−1^ [80]; (**b**) Hydrous ruthenium oxide (RuO_2_) adopted with PAMPS with electrodeposition method and provide specific capacitance of 642 F g^−1^ at 20 mV s^−1^ [81]; (**c**) nanocomposites’ hydrogel electrodes PAMPS/multiwalled carbon nanotubes (MWCNTs) at various weight percentages, providing maximum energy density of 43.8 Wh kg^−1^ at a power density of 520 W kg^−1^ [82]; (**d**) preparation of supercapacitors with the composite of PVA/PAMPS, which provided a capacitance of 156.2 F g^−1^ at temperatures of 80 °C [83]; and (**e**) pure PAMPS.

Poly (2-acrylamido-2-methyl-1-propanesulfonic acid) (PAMPS) and ammonium molybdate [(NH_4_)_2_MoO_4_] (Mo) composite demonstrated an effective supercapactive performance when it is in a redox-mediated environment [77]. Conducting polymers are always combined with other compounds to form complete and compensated properties such as the polyelectrolyte additions; for example, the zwitterionic silica sulfobetaine [78]. This helps to enhance the capacitive performance. The synergic effect with the electrolyte helps to double the capacitance from the pure PAMPS. The use of PAMPS as electrode and electrolyte materials for the preparation of high performance supercapacitor was patented in JUSTIA patents [79]. The work was performed with comprising molybdate (VI) salts dispersed in a hydrogel matrix and claims 360–550 F/g at a current density in a range of 1–10 A/g, which is relatively very high when compared with other similar hydrogel-based electrodes. By combining PAMPS with other polymeric materials such as polypyrrole [80], it was possible to produce a hydrogel electrode with a high capacitance value of 698.8 F/g at 5 mV s^−1^. The work was performed with carbon fibers, which leads to the use of a PAMPS-based hydrogel for the production of high-performance supercapacitors for the use in smart clothing applications. There are several possibilities to form composites with PAMPS so that the mechanical and electrochemical properties are improved for supercapacitor applications. Figure 7 mentions only few of them.

Extensive research has been conducted to uncover the flexible energy storage for wearable electronics. Furthermore, the low cost preparation [84], which is stable at different temperatures [83], is important for wearable applications. PAMPS with a various composition will be the perfect candidate for such applications.

#### 3.3.2. Polyaniline (PAni) Hydrogel

Polyaniline (PAni), an intrinsically conductive polymer, is an intensively studied polymer that is usually produced using oxidative polymerization means [85]. Analine monomers are the sources of PAni. Usually the conductivity of PAni varies based on the dopant variety [86]. By polymerization, PAni-based hydrogels combined with a nonconducting hydrogel matrix has been reported elsewhere [87] for the production of supercapacitor materials with a sufficient electrochemical performance. Conductive polymer-based hydrogels have a unique advantage of being hydrogels and conductive at the same time. Being flexible, biocompatible, hydrophilic and biodegradable, its electroconductivity makes polymer hydrogels perfect candidates for supercapacitor application, which combines with the lightweight and highly integrability of fabrics identified as a promising class of materials for wearable applications. Like other materials, PAni-based hydrogels are most often characterized with its electrochemical properties.

Typically, PAni are combined with other polymers to obtain better electrochemical properties. For instance, PAni has been combined with polyvinyl alcohol (PVA) [88] and has obtained a 237 mF/cm^2^ at current density of 0.5 mA/cm^2^ with excellent charge-discharge cycles. However, there is a difficulty in making a good combination of PVA and PAni; the authors employed a special crosslinking agent to obtain good results. It is also possible to produce supercapacitors with excellent mechanical properties, electrical properties and improved performance in electrochemical properties with PVA-PAni-graphene-based hydrogel composites [89].

Another work performed by Dou, P., et al., [90] combined PAni with amino trimethylene phosphonic acid and achieved an ultrahigh specific capacitance of the specific capacitance over 420 F/g. Amino trimethylene phosphonic acid acts as a dopant and gelator, which helps PAni to increase the porosity and hence the surface area, which is the fundamental quantity that plays to increase the cyclic voltammetry or capacitance of the substance. Actually, the electroconductivity due to the doping surface area because of the gelation addition has been improved by the addition of amino trimethylene phosphonic acid. The use of the hydrogel based on PAni can act as an electrolyte by combining with carboxymethylcellulose and polyacrylamide solution [91], which indicates that PAni-based hydrogels are not only used as electrodes but also electrolytes in supercapacitor production. The final output for the supercapacitor is when it is served as an energy storage for some applications. The production of the wearable sensors were reported using PAni-based hydrogels as supercapacitors [92]. The authors in this work claimed that the production of the solid electroless in the preparation of supercapacitors with a sandwich structure obtained a real capacitance of 635 mF/cm^2^ with hydrochloric acid as a dopant to increase the electrical conductivity. Therefore, supercapacitors produced from PAni-based hydrogels are feasible for the production of wearable electronics.

#### 3.3.3. Poly(3,4-ethylenedioxythiophene) Polystyrene Sulfonate-Based Hydrogel

Poly(3,4-ethylenedioxythiophene): polystyrene sulfonate (PEDOT:PSS) is a well-researched intrinsically-conductive polymer due to its high electromechanical properties [12]. PEDOT:PSS has received attention not only due its electrochemical properties but also its water dispersibility, ease of production, environmental stability and easy availability [93]. Similarly, PEDOT:PSS is water dispersible, as the responsible polystyrene sulfonate that makes PEDOT water dispersible; it decreases the electrical conductivity. Doping with ionic solution will help to move the hydrophilic PSS towards the surface after the water dispersion occurred [12]. This property of PEDOT:PSS makes the polymer a perfect candidate for the formation of electroconductive hydrogels by combining other polymeric compounds. For instance, the PEDOT:PSS-based hydrogel with the AlCl_3_-induced cross-linking was prepared using fast gelation principle and obtained a capacitance of 158 F/g at a scan rate of 50 mV/s with 84.9% capacitance retention after 2000 cycles. Ethylene glycol has been used an ionic dopant element to shift the PSS part of the conductive polymer. Table 3 shows the comparisons of the supercapacitor based on the PEDOT:PSS hydrogels. Current density (A/g; mA/cm^2^), energy density (µWh cm^−2^) and power density (mW cm^−3^) values are incorporated for comparison purposes.

The conductive hydrogel-based PEDOT:PSS has received considerable attention for the production of flexible electrodes due several reasons such as flexibility, eco-friendliness, low production cost and excellent mechanical strength [104,105]. These properties make PEDOT:PSS-based hydrogels to be selected for the applications of wearable electronics. For instance, self-healable and stretchable hydrogels [106], fiber hybrid-based with excellent volumetric capacitance [104], and conductive hydrogels [27] have been investigated for the use of energy harvesting for wearable electronics. Generally, supercapacitors for the energy harvesting-based electrically conducting PEDOT:PSS polymer have been given recent attention for various applications such as wearable electronics due to their light weight and electrochemical performance properties. Sometimes, polyvinyl alcohol (PVA) has been used as electrolyte to increase the supercapacitor performance [107].

#### 3.3.4. Polypyrrole (PPy)-Based Hydrogels

Polypyrrole (PPy) is a similar kind of intrinsically conducting polymer with excellent electrical properties. However, since it is a п-electron conjugated polymer type, it is brittle and limits its practical use, especially for flexible electronics [108]. Therefore, the common way of preparing the PPy-based hydrogels is making composites to compensate its poor mechanical properties. For instance, Das, D. and S. Kurungot, [109] reported the gelation initiated by the cross-linking of the dopant 5,10,15,20-tetrakis (4-sulfonatophenyl)-21H,23H-porphine manganese (III) chloride (MnTSPP) anion in the PPy chains, that is able to produce flexible supercapacitors that can be further used in the production of wearable clothing for various health-related applications. Another means to overcome such a poor mechanical property is wrapping PPy onto graphene-based hydrogels [110]. The hydrogel based on PPy-graphene achieved high performance supercapacitors with large specific capacitance, good rate capability and cycle stability. This is because graphene is a 3D architecture with high surface area, which helped to improve the poor mechanical property of PPy. When PPy is presented in a hydrogel form, poor mechanical properties can be improved. For instance, the solid-state high performance and flexible supercapacitor was prepared using the PPy hydrogel [111]. The high mechanical property with excellent elongation and flexibility makes easier use of PPy-based hydrogels for the production of wearable clothing.

The environmentally friendly process, low manufacturing cost, flexibility, lightweightdness and ultrahigh porous structure are required and preferred for the manufacturing of energy storage supercapacitors. This issues are currently realized by the combination of bio-based materials suing the lignosulfonate/polypyrrole (Lig/PPy) hydrogel (LP54) [112]. However, electromechanical performances are weak, excepting some enhancement mechanisms that are carried out. Sometimes, without the additions of any cross-linkers, it was possible to create supercapactive PPy-based hydrogels by controlling its morphology [113]. PPy was less used in the production of supercapacitors in its hydrogel form when compared to the other conducting polymer hydrogels discussed in the review. It was able to achieve acceptable electromechanical performance supercapacitors, which can be easily scalable and further used for the production of wearable clothes.

### 3.4. Cellulose-Based Hydrogels for Supercapacitor Applications

**Figure 8:**
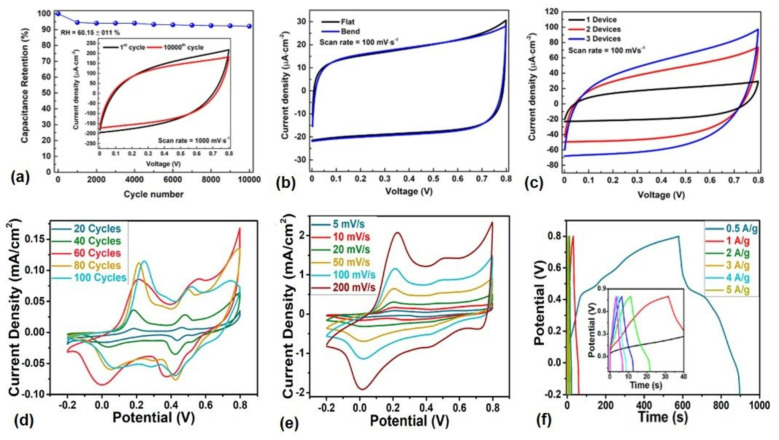
Performance of supercapacitor hydrogel materials from cellulose and its composites. (**a**) the performance of microcrystalline cellulose (MCC)-based supercapacitors under scan rate of 1000 mV/s for 104 CV cycles; (**b**) the CV of the device fabricated based on MCC in bent and flat positions; (**c**) the CV curves of the device at 100 mV/s constructed in parallel; (**d**) CV curve for cellulose based (cellulose from balsa wood) with various deposition cycles at 5 mVs^−1^; (**e**) CV curves of the composite hydrogel with 60 deposition cycles at 5–200 mVs^−1^ composite hydrogels; (**f**) charge-discharge curve of cellulose-based composites using galvanometry. Subfigures (**a**–**c**) are re-printed with permission under open access license under terms and conditions of the CC BY-NC-ND 4.0 with Ref. [117]; (**d**–**f**) re-printed with permission under open access license under terms and conditions of the CC BY 4.0 with Ref. [118].

The major commercial materials for the production of supercapacitors mainly depend on carbon and carbon-based compounds in the activated carbon form because of their high surface area due to its porous structure [114]. Most recently, cellulose-based hydrogels have also been paid some attention [115]. Even though the base material is cellulose (bamboo), the basic principle behind it is changing the cellulose into activated carbon [116]. Recent advances in the field of hydrogel preparation for supercapacitor applications have been focused on the use of cellulose-based hydrogels due to their outstanding properties. Figure 8 shows the performance in terms of the electrochemical properties of the supercapacitors produced, using cellulose as a base material.

Producing a sustainable energy storage is the demand of future wearable electronics. In this regard, hydrogels from cellulose materials have demonstrated a permissible approach. The performance obtained from cellulose and cellulose-based composite hydrogels demonstrates an indicative that these categories can replace the fuel-based energy storage systems. In addition to the performance, the stability under certain conditions is also important. Figure 8a) shows that the device constructed from cellulose-based hydrogels can sustain up to 92% after 104 CV cycles. The durability of the supercapacitor materials under different cycle and bending conditions is also very critical for the charge-discharge performances (Figure 8b,d). The cyclic voltammetry measurement is the fundamental quantity to measure the electrochemical performance of the supercapacitors, and the cellulose-based hydrogels demonstrated a high performance in this regard (Figure 8c,e). Quick charge-discharge is necessary for any supercapacitor materials [119]. Cellulose-based supercapacitors demonstrated this property (Figure 8f), which is very necessary for the wearable electronics’ construction. Therefore, it is possible to construct high performance supercapacitors with composite hydrogels based on cellulose materials that are light weight, flexible and stretchable, which can be deposited onto textile substrates and help to manufacture energy storage materials for smart textiles.

The contribution of cellulose-based materials for the sustainable environment is very important. Attributed to this fact, the lignin-based biodegradable material based on electroconductive hydrogels is among them [120]. Lignin has demonstrated a high specific capacitance of 298.6 F/g at 10 mV/s) and excellent rate performance, bringing a high energy density of 13.7 Wh k/g and outstanding capacitance retention of 89.9% after 10^4^ cycles [121]. The report in Ref. [122] demonstrated an excellent electrochemical performance when other materials make composites with cellulose. Converting the cellulose materials into carbon materials is an important step to obtain the best conductivity [123] and hence apply as an electrode in the supercapacitor device fabrication.

### 3.5. Other Hydrogels for Supercapacitor Production

There are also other hydrogels that have been used for the production of supercapacitors and in the assembly of wearable clothes. In this section, some other hydrogel materials that have been used in the production of supercapacitors for wearable clothes are discussed.

#### 3.5.1. Chitosan-Based Hydrogels

Chitosan is a biocompatible, biodegradable and hydrophilic natural cationic copolymer [124]. Chitosan is a unique bio-polymer, and it is possible to obtain gels out of it using different means such as the physical/chemical reticulation process (bond formation) and gels without any crosslinkers (such as the acetylation process) [125], which helps to prepare hydrogels with various functions. Even though the best applications for chitosan are potentially the engineering scaffolds that obtain tissue repair achievements, nowadays, the use of chitosan as an active electrode for the preparation of a supercapacitor is also obtained as an equivalent attention. For instance, the authors in Ref. [126] prepared chitosan-based hydrogels in combination with Li^+/^Ag^+^ and achieved an aerial capacity of 1 mF cm^−2^ at a 1.8 mA cm^−2^ current density, which can survive for more than 10,000 cycles without losing its capacitive performance. Efficient supercapacitors can be produced using chitosan hydrogels [127]

As stated above, the chitosan-based hydrogels can be formed either by the use of external crosslinkers or without using such crosslinkers. Yang, H. et al., [128] reported the crosslinking of chitosan using HCl and obtained a capacitance of 45.9 F g^−1^, energy density of 5.2 Wh kg^−1^ and power density of 226.6 W kg^−1^, and realized the solid-state supercapacitor using a phase separation means. The electrolyte uptake and conductivity of the transparent film was super high. In addition to crosslinking with other biopolymers, the modification of the chitosan to form hydrogels was also achieved [129]. Yang, H. et al., prepared a modified carboxylated chitosan synthesized through a free radical graft copolymerization of acrylamide monomers and chemical crosslinking principles to obtain higher ionic conductivity, and produced an electric double layer capacitor (EDLC) with excellent performance.

For wearable applications, flexible supercapacitors are required. Flexible supercapacitors with a lightweight and simple film structure have been reported elsewhere [130]. The flexible supercapacitors were prepared by using a blend of the chitosan/graphene@ (manganese carbonate nanoparticles) MnCO_3_ polymer hydrogel and obtained a specific capacitance value of 312.5 F g^−1^. In addition, the PVA/quaternary ammonium–chitosan hydrogel electrolyte was prepared for detection supercapacitors with brilliant performance [131], which can be attached to the wearable clothes for sensing applications. The chitosan-based oxygen-doped activated carbon/graphene composite for flexible supercapacitors was also reported [52]. This kind of supercapacitor for portable energy storage purposes can be integrated with wearable clothes for energy sources. Overall, the chitosan-based hydrogels are gaining recent attention for the making of flexible, portable and high-geared supercapacitors. These kinds of supercapacitors have the capability to be integrated with wearable clothing for smart textile applications. The other reason why chitosan-based hydrogels are gaining attention is due to its biodegradability, biocompatibility, eco-friendly nature and easy accessibility from natural resources such as plants, animals and other natural resources [132]. Therefore, it is highly recommendable to invest in and utilize chitosan-based hydrogels for supercapacitor production and other similar purposes.

#### 3.5.2. Sodium Alginate

Hydrogels with a electrically conductive nature are being highly explored in the fabrication of flexible and portable energy storage devices due to their excellent electrochemical properties. Sodium alginate hydrogels provide such an excellent electrochemical conductivity when doped with conductive polymers such as polypyrrole [133], reduced graphene oxide (rGO) [134] and Ag nanoparticles [135], which exhibited an excellent electrochemical performance and are able to prepare high performance supercapacitors. It was possible to obtain a high capacitance, excellent rate capability and good cycling ability with the sodium alginate-based hydrogels. Furthermore, flexible supercapacitors using chitosan/sodium alginate composite hydrogels has been reported [136], which are likely to be used for wearable clothing applications. Sometimes, the binder may not be required, and using the physical crosslinking can help in obtaining highly conductive sodium alginate hydrogels [59]. Strain sensors with flexible supercapacitor production approach was claimed in Ref. [137]. Such kinds of supercapacitors are highly stretchable and self-healable, which can be an excellent potential for wearable clothing. All the other information guided us to understand that highly conductive sodium alginate hydrogels are capable of producing highly geared supercapacitors for wearable clothing applications. Composites of sodium alginate and rGO are able to provide a capacitance of 753 F. g^−1^ at 1 A. g^−1^ with a good rate capability and cycling stability up to 5000 cycles [134].

#### 3.5.3. MXene-Based Hydrogels

**Figure 9:**
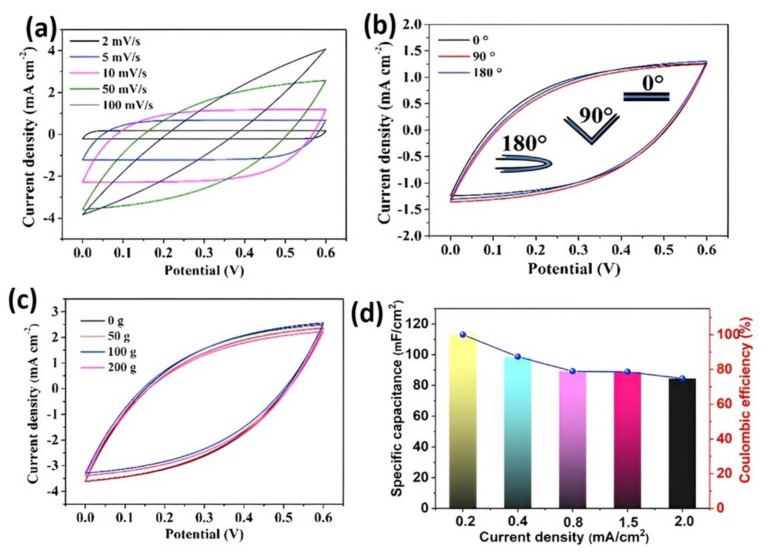
Electrochemical performances of MXene-based hydrogels using PVA/carboxymethyl cellulose film electrolyte. (**a**) Cyclic voltammetry curves at various scan rates; (**b**) cyclic voltammetry curves at various bending angels at a scan rate of 10 mV/s; (**c**) cyclic voltammetry curves at a scan rate of 10 mV/s; (**d**) capacitance at various current densities. Re-printed with permission under the Creative Commons CC-BY-NC-ND @ Cell Reports Physical Science 3, 100893, 18 May 2022 @ 2022, The Author(s) [139].

MXene is a group of two-dimensional inorganic substance with high electron density, which can be predicted to be metal except for its hydrophilic properties due to the hydroxyl- or oxygen-terminated surfaces [138]. Its high conductivity might be used where a high electrochemical property is required. MXene-based hydrogels have been used in the preparation of flexible supercapacitors and possess excellent electrochemical performance [139]. Figure 9 shows the electrochemical performances of MXene-based hydrogels. Hydrogels based on MXene are often used in the production of supercapacitors for wearable applications due their flexible nature [140].

Both PVA and carboxymethyl cellulose owe their excellent mechanical properties to the composite hydrogels, which benefited from them to produce the highly flexible supercapacitors. The excellent performance in the potential versus current density curve (Figure 9a) indicates that the MXene-based hydrogels possess satisfactory pseudocapacitance characteristics of the composite materials. The cyclic voltammetry curves indicate that an overlapping during bending (Figure 9b) and during different loadings (Figure 9c) demonstrates an outstanding flexibility and retain their capacitance after several bending and loading actions.

To increase the conductivity of the hydrogel, MXene has been combined with graphene as a gelation element and obtained a superhigh gravimetric and volumetric capacitance [141]. For wearable clothing applications, high conductivity is not sufficient, rather flexibility and light delightedness are equally important. Flexible supercapacitors based on MXene-based hydrogels composites were made possible [142].

## 4. Challenges of Hydrogels for Flexible Supercapacitors

Even though the progress in the development of flexible hydrogels demonstrated an excellent improvement, there are still several problems, which remain a challenge. One of the challenges is the selection of the electrolyte, i.e., obtaining a nonflammable, noncorrosive, biocompatible and safe electrolyte [143]. Organic electrolytes have the above-mentioned problems. Ren, M., J. Di, and W. Chen reported that the ability of self-charging and low self-healing properties of wearable supercapacitors are still a challenge. Self-aggregation, low mechanical properties and limited functionality are the drawbacks of lignin-based hydrogels for supercapacitor applications [120]. Most of the hydrogels must form composites to compensate for such problems. However, a more complicated and long process of composite formation is also a challenge for wearable supercapacitors from hydrogels. Peeling off and loss of conductivity are by the far the most critical problems of hydrogel conductive materials. This is because when the device is flexing, the cracking of the conductive track may lose, and the hydrogels sometimes peels off from the substrate due to adhesion problems. In addition, the polymer binders can solve this but simultaneously it will reduce the conductivity of polymer hydrogels.

The stability against the environment, for instance from a high temperature, still remains a challenge for hydrogels [144]. In addition, oxidative stability, cyclic performance and charge-discharge ability are the basic problems [145]. While lots of progress has been performed to create flexible supercapacitors in recent times, it is still an ongoing process to enhance the energy and power density of the supercapacitors.

## 5. Summary and Outlook

Supercapacitors are receiving recent attention in the field of energy storage device production. This is because supercapacitors provide high energy density, excellent charge-discharge performance, flexibility and long-life cycle. Furthermore, flexible supercapacitors have the capability to provide wider options in terms of portability, stretchability and light weight properties. In this regard, wearable clothing requires highly demanding portable energy storage devices for various applications. Flexible supercapacitors are very promising innovations for wearable clothing applications, which require high capacitance, high power ratings, long life cycle, stretchability, portable appearance and durability. Those requirements are realized by sustainable energy systems using hydrogel materials. In this review, different hydrogels materials as electrolyte and electrode components for flexible supercapacitor applications were discussed, pointing out their capacitive performance, energy density and power density. Hydrogels that have been used in the production of flexible supercapacitors for a wearable application were given particular attention. The review pointed out that optimization in the formation of hydrogels were crucial for the maximum performance of the supercapacitor. A proper selection of composite materials against the base of conductive hydrogels should be given special attention for electrochemical and mechanical property optimizations. Apart from the hydrogel formation, the design and configuration of the supercapacitor will gear equal contribution for the optimum supercapacitor capability. The review results depict those differences in the energy density, and the power density provides a difference in the specific capacitance value; hence, the difference in the electrochemical performance of the supercapacitors. Finally, the importance of the process electrodes and electrolytes should be optimized to maximize the device performance.

## Figures and Tables

**Table 1:** Comparison of supercapactive performance of graphene-based hydrogels.

Composite Materials	Methods	Capacitance Value	Ref.
Hydrazine hydrate	Chemical oxidation	190 F/g at 0.5 A/g	[48]
Chitosan	Microwave-assisted hydrothermal	377 F/g at 5 A/g	[51]
Chitosan	One-step hydrothermal method	375.7 F/g at 1 A/g	[52]
Ethylenediamine	Two-step hydrothermal method	−240 F/g at 1 A/g	[45]
Carbon nanotube	Chemical reduction	10.13 mF/cm^2^	[53]
PVA/Zn/EG	Chemical dissolution	247.7 F/g at 5 A/g	[54]
PVA	Microwave-assisted cross-linking	163 F/g at 1 A/g	[55]
Potassium acetate	Hydrothermal	55.1 F/g at 0.3 A/g	[56]
Polyaniline	Polymerization	500.13 mF cm^−2^	[57]
Chitosan	In situ ion cross linking	107.6 F/g at 1 A/g	[58]
CuS/ZnS/sodium alginate	Physical crosslinking followed by one-step reduction	252.1 F/g at 5 mV/s	[59]
Ti_3_C_2_Tx MXene	Gelation process	26 F/g at 1 V/s	[60]
NiCo oxide	Coagulation-induced self-assembly	858.3 F/g at 2 A/g	[61]
CoFe_2_O_4_	In situ via a facile one-pot solvothermal approach	356 F/g at 0.5 A/g	[62]
Polyampholyte	One-step random copolymerization	216 F/g at 0.5 A/g	[63]
β-cyclodextrin	Hydrothermal reduction	310.8 F/g at 0.5 A/g	[64]

**Table 2:** Comparisons of supercapactive performance of CNT-based hydrogels using various composite materials.

Composite Materials	Methods	Capacitance Value	Ref.
Cellulose	Induced polymerization and cross-linking	1786 mF/cm^2^ at 1 mA/cm^2^	[67]
Graphene	Aerosol chemical vapor deposition	806 F cm^−3^ at 112 mWcm^−3^	[68]
ZnNiCo hydroxide/graphene	Catalytic carbon vapor deposition	1.185 mAh cm^−2^ at 5 mA cm^−2^	[69]
Nanocellulose/PVA	Dissolution	117.1 F g^−1^ at 0.3 A/g	[70]
Nanocellulose	Oxidation	65 F g^−1^ at 0.4 A/g	[71]
Polyaniline	Graft polymerization	880 F g^−1^ at 1.5 A/g	[72]
Carboxymethylcellulose-polyaniline	Layer-by-layer assembly	3106.3 mF cm^−2^ at 5 mA cm^−2^	[73]
Polyaniline	In situ chemical oxidation	647 F g^−1^ at 1 A/g	[74]

**Table 3:** Comparison of supercapactive performance of PEDOT: PSS-based hydrogels.

Composite Materials	Methods	Capacitance Value	Ref.
Polyvinyl alcohol (PVA)	Solution immersion	128.9 mF cm^−2^ at 11.46 µWh cm^−2^	[94]
Bis(trifluoromethane) sulfonamide lithium Salt (LiTFSI), PVA	Novel incorporation	44.5 mF cm^−2^ at 0.04 mW cm^−2^	[95]
Molybdenum disulfide	Hydrothermal process	360 mF cm^–2^ at 0.5 mA cm^−2^	[96]
Aluminiumchlorid	Induced cross-linking	158 F/g at 0.6 A/g	[97]
Poly (acrylamide)	Free radical polymerization	327 F/g at 1 A/g	[98]
PVA/poly (methacrylic acid)	Polymerization	7.38 mF cm^−2^ at 10 mA cm^−2^	[99]
H_2_SO_4_	Thermal treatment	202 F cm^−3^ at 0.54 A cm^−3^	[100]
Polyaniline	Polymerization	112.6 F/g at 0.25 mWh cm^−3^	[101]
Polyaniline	Thermal process	808.2 mF cm^−2^ at 0.63 mWh cm^−3^	[102]
MWCNT	Thermal treatment	485 F/g at 1 A/g	[103]

## Data Availability

Not applicable.
